# A German Smartphone-Based Self-management Tool for Psoriasis: Community-Driven Development and Evaluation of Quality-of-Life Effects

**DOI:** 10.2196/32593

**Published:** 2022-07-07

**Authors:** Lea C Brandl, Claudia Liebram, Wendelin Schramm, Monika Pobiruchin

**Affiliations:** 1 Institute of Telematics University of Lübeck Lübeck Germany; 2 Consumer Health Informatics SIG German Association for Medical Informatics Biometry & Epidemiology (GMDS e.V.) Cologne Germany; 3 Psoriasis-Netz Berlin Germany; 4 GECKO Institute for Medicine, Informatics & Economics Heilbronn University Heilbronn Germany

**Keywords:** psoriasis, self-management, mobile apps, quality of life, mobile phones, smartphones

## Abstract

**Background:**

Psoriasis is a chronic disease characterized by inflammation, increased scaling, itching, and other symptoms. Psoriasis is not contagious, but patients have often felt shunned. Therefore, in addition to psoriasis symptoms, stress, anxiety, and depression can also affect quality of life (QoL). Surveys show that only a quarter of patients are satisfied with the success of their therapy. However, in addition to medical therapy, self-management can also make it easier to deal with chronic diseases like psoriasis.

**Objective:**

The aim of this project was to develop a smartphone-based self-management tool (SMT) specifically for patients with psoriasis using a community-driven process. The impact of the SMT on QoL as well as its acceptance and usability were evaluated.

**Methods:**

In collaboration with an internet-based self-help community, 2 user surveys were conducted to determine the requirements for a smartphone-based SMT. The surveys consisted of semistructured questionnaires asking for desired features in an SMT for psoriasis. A pilot study was conducted to evaluate QoL, acceptance, and usability. Community users were recruited to use the app for 21 days and complete the Dermatology Life Quality Index (DLQI) questionnaire at the beginning (T_0_) and end (T_1_). Afterward, participants were asked to complete another questionnaire on usability and ease of use.

**Results:**

SMT requirements were collected from 97 members of an internet-based community. The SMT was built as a progressive web app that communicates with a server back end and an Angular web app for content management. The app was used by 15 participants who also provided qualitative feedback, and 10 participants answered all questionnaires. The average DLQI score was 7.1 (SD 6.2) at T_0_ and 6.9 (SD 6.6) at T_1_. The minimal required sample size of 27 was not reached.

**Conclusions:**

The high degree of community participation in the development process and the responses during the requirement engineering process indicated that there is a general need for an independently developed SMT for patients with psoriasis. However, the feedback received after app use shows that the SMT does not meet the needs of the community. It can be concluded that a more customizable app is needed. The focus and needs of the users were very heterogeneous. Similar developments and research could benefit from the findings of this project.

## Introduction

### Overview

Psoriasis is a chronic skin disease that manifests itself with inflammation, increased scaling, itching, and other symptoms [1]. Flares can be aggravated by stress, medical drugs, or infectious diseases [2-4]. Psoriasis affects approximately 2% to 3% of the German population [1,5]. In addition to psoriatic symptoms, stress, anxiety, and depression can affect quality of life (QoL) [6-8]. Compared to healthy individuals, anxiety disorders were identified more frequently in patients with psoriasis [4,9]. It is not possible to cure psoriasis, but symptoms could be mitigated with medical drugs, special therapies, or complementary methods [10,11]. Nevertheless, approximately 30% of patients do not consult a physician [12,13].

Interviews reveal that a quarter of patients are dissatisfied with the success of their treatment [14,15]. Problems regarding drug therapies are present in up to 40% of patients [15,16]. To increase patient satisfaction and adherence, evidence-based decision guidance for psoriasis therapy is available as an S3 guideline [17]. However, in addition to medical therapy, self-management can facilitate the management of chronic diseases such as psoriasis [18-20]. In comparison to other chronic diseases, there are less evidence-based self-management tools (SMTs) for patients with psoriasis [20].

### Related Work

Self-help and self-management can be effective tools for patients to better cope with their (chronic) disease [18-20]. Traditionally, self-help involves in-person group meetings or counseling. In recent years, a shift toward mobile apps, information websites, and web-based communities can be observed. Self-help can also include aspects of citizen science approaches. For example, self-help groups and other organizations collect and organize information on people with a disease [21]. Internet-based communities are often used by patient experts who share information and generate hypotheses. This is done based on shared experiences. Modern technologies can facilitate new approaches to citizen science projects [22].

Citizen science also includes the involvement of patients in the development process of SMTs. Some examples are given in the following paragraphs. Safdari et al [23] conducted a study on the requirements for a self-management app for patients with psoriasis. In a requirements analysis, 100 patients provided information about their requirements for educational information and lifestyle management, among other features. This included information on the disease on the one hand and factors such as physical activity, nutrition, or stress management on the other hand.

Trettin et al [24,25] have developed an app for Danish patients with psoriasis treated with biologics. In the development process, patients were interviewed, and various workshops and a prototype test were carried out. In the app, vital signs, and the Dermatology Life Quality Index (DLQI) [26] can be recorded in preparation for video or telephone consultations. Patients, doctors, and nurses have reported that consultations are more structured, and patients feel safe [24,25].

### Aims of the Study

Currently, there are few SMTs in German app stores and no German-speaking apps (co-) developed by self-help organizations (ie, the patients themselves). Therefore, the 2 major objectives of this study are to (1) present an SMT especially developed by and for patients with psoriasis and (2) evaluate the app’s impact on QoL as well as its acceptance and usability.

## Methods

### Recruitment and Requirement Engineering Phase

In Germany, there are 2 main organizations for psoriasis self-help. The larger one is the Deutscher Psoriasis Bund e.V. (German Psoriasis Association) and the smaller one is the Psoriasis Selbsthilfe Arbeitsgemeinschaft e.V. (Psoriasis Self-Help Association, PSOAG) [27,28]. The latter offers self-help on its website. The internet-based community consists of approximately 28,000 users (as of November 2019) and concerns itself with topics such as therapies or nutrition. There are various expert forums and groups that can be joined. Knowledge articles are also published [29]. The project was initiated with PSOAG members. The community collected requirements of a (potential) smartphone-based SMT during 2 online surveys (published via the web-based forum).

The first survey started on February 2, 2019, and was closed on March 15, 2019. It consisted of a semistructured questionnaire that asked about the desired features of an SMT for psoriasis. A translated version of this questionnaire can be found in Multimedia Appendix 1. After completion of the first survey, the responses were read and clustered to capture different categories of functionalities. In the next step, categories were checked for technical feasibility and focus on SMTs.

The second survey that incorporated the community feedback from the first round was open from June 25, 2019, to August 31, 2019. During this phase, 11 mock-ups of the SMT were presented and discussed with the community members. The mock-ups were built with the Balsamiq Mockup software (version 3.5.17; Balsamiq Studios, LLC) [30].

Changes needed in the design of the app and the expansion of certain functionalities were again checked for technical feasibility and incorporated into the final app requirements.

After the requirements were determined, the SMT development started. The system was implemented by one of the authors using software components previously developed [31] as blueprints. The app development and the pilot study were carried out as part of a master's thesis at Heilbronn University.

### SMT Evaluation

#### QoL Assessment

Numerous instruments of QoL assessment exist [32,33]. Fitzpatrick et al [34] developed a list of criteria to select the most appropriate measurement instrument. According to these criteria, we decided to use the DLQI by Finlay et al [26] for this study. The DLQI consists of 10 questions that cover the dimensions of symptoms and daily activity, leisure, work or school, personal relationships, and therapy [26]. The average response time is 2 minutes [35]. The score of the DLQI ranges from 0 to 30. The larger the value, the worse the QoL [26]. The minimal clinically important difference is 4 score points [36]. A license for the use of the German Translation DLQI was applied for and was provided by Cardiff University [35].

#### Use and Usability

Considering usability and further use, an additional questionnaire was introduced. It included questions about handling, use, further use, and the severity of psoriasis, and were self-reported and/or measured by the Psoriasis Area and Severity Index (PASI) [37]. This additional questionnaire can be found in Multimedia Appendix 2.

### Recruitment for QoL Assessment

Participants for the QoL assessment were recruited through various channels:

1. A call for participation was given via the PSOAG website and forum. The call provided the opportunity to contact the study team directly or to access a study information website.
2. Information flyers were sent to dermatology clinics (13 rehabilitation clinics and 11 university clinics), the Professional Association of German Dermatologists, and the Psoriasis Association.
3. Social media channels were used to actively promote the study (eg, regular tweets, posts on Instagram, and the creation of a Facebook page about the study).

For inclusion in the study, the participants had to have psoriasis and be at least 18 years old.

### Study Design

As the study measured the change in QoL, it was necessary to record it at a minimum of 2 time points (T_0_ and T_1_). The DLQI measures QoL in relation to the last 7 days. A break of at least 7 days is recommended, and frequent questioning is discouraged; otherwise, participants may remember their previous response. Based on these requirements, the intervention duration was set at 21 days.

After 21 days had elapsed, the second DLQI and the extra questionnaire on usability and further use of the SMT were completed. Then, the participants dropped out of the study.

### Statistical Analysis

Based on the German-specific data provided by Lesner et al [7] and the minimal clinical effect of 4 points, a sample size of 27 participants is calculated to reach *α*=.05 and *β*=.2 [38]. Data were analyzed with the statistical software MATLAB (version 2019b) [39].

### Ethical Approval

No ethical approval was obtained for the conducted study. The local ethics committee of Heilbronn University is only an advisory board (see §1(1) [40]), and it does not provide formal ethical approval. This was a community-led project involving interested persons with psoriasis who enrolled voluntarily and by general invitation in the development and evaluation of an app. These were not patients treated by the authors. Treatment changes or any interaction with the medical care delivery team were not included in the objectives of this study.

As the data collected were worthy of protection, a high scientific standard was applied here. All participants were informed about the study details and received written information in accordance with the Declaration of Helsinki [41]. This information was available on the study information website and could be downloaded for offline reading (see Multimedia Appendix 3). All participants had one-to-one contact with the principal investigator and questions from them were answered by email or telephone, as requested.

All participants signed an informed consent form (see Multimedia Appendix 4), which could be revoked at any time. In this case, all documents that could still be assigned were destroyed. To ensure a high level of data security, the questionnaires were recorded under a pseudonym generated by the participants themselves.

## Results

### Requirement Engineering and SMT

More than 90 community members participated in the survey (N=97). They determined the scope of the SMT’s features, created the medical content, and curated it.

During the first user survey, the requirements for the SMT were collected. Nearly 90% (87/97) of the participants indicated willingness to test the SMT. The suggestions of the community about the desired features were evaluated for their feasibility and purpose of use. Four categories emerged during the analysis of the community responses: (1) communication, (2) drug management, (3) tracking of complementary methods, and (4) rate/score doctors. Selected examples of the feedback from the SMT users are presented in Table 1. Furthermore, mock-ups (ie, drafts of the user interface) were created. These were presented to the community and the community members could provide their comments.

**Figure 1:**
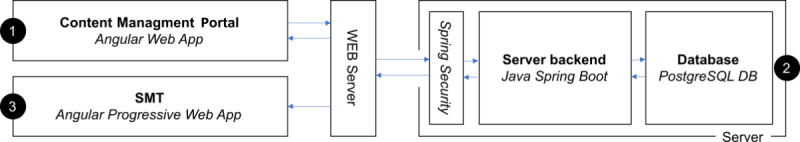
System architecture. The 2 Angular components are shown on the left. These are provided on a web server that communicates with the back-end server. A Spring Security module protects the back end and the connected database from security attacks. The back-end component is shown on the right. DB: database; SMT: self-management tool; SQL: structured query language.

The final SMT framework consists of three components, as shown in Figure 1: (1) an Angular web application for the management of the SMT content used by the editorial team, (2) a server back end built with Java Spring Boot and Spring Security, and (3) the SMT for the study participants as an Angular application. The SMT app was implemented as a progressive web app (PWA) to be available for all types of smartphones.

Both web components were built with the Angular Framework (Google; version 7.2.15) [42]. The server back end was built with Java Spring Boot (The Spring Team; version 2.1.5) [43] and Java (Oracle Corporation; version 11) [44].

One feature focus of the SMT is to suggest interaction-free complementary measures to the user, with which physical complaints, such as itching, skin blisters, and dry skin, can be alleviated. Patients can document their psoriasis type, medical drugs, and complaints each day. Based on these data, the SMT suggests complementary methods that are free of interactions. The complementary measures were suggested by the community itself and were reviewed by the forum's editorial team. In total, 55 complementary measures were found, described, and incorporated into the knowledge base of the SMT (see component 1 in Figure 1). The complete list is available in Multimedia Appendix 5. The knowledge was embedded in a PostgreSQL database (PostgreSQL Global Development Group; version 10.9) [45], as shown in Figure 1.

**Figure 2:**
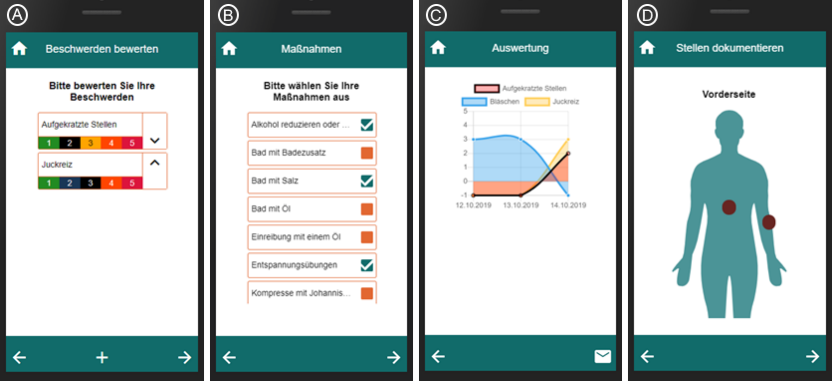
Screenshots of the smartphone app. Left to right: (A) Rating and sorting physical complaints. Complaints can be added via the “plus” sign in the footer. (B) Choosing complementary methods, including reducing alcohol, taking a bath with bath salts, and relaxing. (C) The course of complaints. By clicking on one of the dots in the chart, the complementary methods used during this day are shown. (D) Documentation of the front of the body. A click on the body adds a region of interest. Photos and text could be documented for each region of interest.

Another aspect of the app was the documentation of psoriasis and the possibility to view the severity of symptoms as they progressed and in relation to the complementary methods that were considered. Moreover, individual body parts could be documented in more detail with the help of photos and text. Figure 2 shows representative screenshots.

A comprehensive data protection concept was developed. All the app data were stored exclusively on the user’s own smartphone.

**Table 1:** Results of the first requirement analysis involving the web-based community^a^.

Feedback from the community	Category	Realization in the SMT^b^
“Input of laboratory values and their graphical representation in order to be able to observe their development (eg, CRP^c^, leukocytes, lymphocytes)”	2	—^d^
“Alarm clock to remind of injection days, medication intake, doctor’s appointments”	2	—
“One should be able to print out what has been written”	3	✓^e^
“Being up to date on care for scalp psoriasis - nutrition tips, new findings”	3	+^f^
“Diet plan, natural remedies”	3	+
“Exchange with other patients (ie, link to the forum)”	1	—
“Notifications about new findings regarding the therapy used (side effects, new variants)”	2	—
“All [measures] that improve my quality of life, take away pain, and build me up”	3	+
“It could remind me to take medication”	2	—
“Study situation and results on complementary therapy methods would be good”	3	✓
“Some kind of daily checklist to check off to-dos related to (psoriasis] would be great. That way you can see correlations, if necessary (eg, if you forgot a supplement or only applied cream once a day instead of twice).”	3	+
“Could you name and comment on current dermatologist (recommend, yes/no and why)”	4	—
“That you can print out the diary would be good to present to the doctor who treated you!”	3	✓
“A list of my current medications as well as a list of medications I have tried but no longer use (and why!)”	2, 3	+
“A chat or a link to a forum where users can exchange information directly. Because I see the main problem with the app as being that something different helps everyone. It’s so hard to generalize.”	1	+
“Calendar in which one can enter appointments for the doctor, taking one’s medication or other things.”	1, 2	—
“Logging what you have eaten and then automated evaluation of whether patterns are recognizable, how nutrition affects you. The same with stress/well-being.”	3	✓
“Maybe refer to competent doctors, dermatologists and rheumatologists in the respective federal states of the people concerned.”	4	—
“To find other patients with psoriasis nearby and find experts or good doctors from the patient’s point of view.”	1, 4	—
“A digital (medication plan) for all medications, not only for psoriasis treatment; in my case, for example, this was accompanied by a CHD^g^ disease.”	2	—

^a^All statements were translated from German. Additions or replacements for better understanding are denoted in square brackets.

^b^SMT: self-management tool.

^c^CRP: c-reactive protein.

^d^The function is not implemented.

^e^The function is fully implemented.

^f^The function is partially implemented.

^g^CHD: congenital heart defects.

### SMT Evaluation

#### QoL Analysis

During the call for participation, 29 persons showed interested in participation, with 21 of them providing consent to the processing of their data. Of these, 18 submitted the initial questionnaires. All persons who returned the first questionnaire are counted as participants. Participants who revoked consent (3 participants) or did not return the second questionnaire (5 participants) are counted as dropouts. Only 10 participants returned both questionnaires that were subsequently analyzed.

In total, 15 valid preintervention and 10 postintervention QoL questionnaires, and questionnaires about acceptance and usability were collected and transcribed into a CSV format. Additionally, 6 participants did not return any questionnaires but gave consent.

The mean DLQI for the preintervention questionnaire was 7.1 (SD 6.2) score points and 6.9 (SD 6.6) score points for the postintervention questionnaire. A lower score after the intervention corresponds to an increased QoL. However, the reduction of the DLQI by 0.2 score points does not correspond to a clinically relevant effect.

#### Use and Usability

The survey on difficulties in using the app shows that users did face challenges (see Table 2).

The results showed that 4 participants had no difficulties in using the app, and 3 participants found it quite or very difficult to use. Moreover, 3 other participants had slight difficulty in using the app. This was also reflected in the feedback from the participants. When returning the second questionnaire or withdrawing from the study, there was additional qualitative feedback on the SMT given by various participants, which is documented in Textbox 1.

The participants were also asked about their continued use of the app. None of the participants would continue to use the app daily. Only 2 participants continued to use the app regularly, 1 participant used it as needed, and 6 participants did not continue to use the app at all. In 1 questionnaire, this question remained unanswered.

**Table 2:** Study results^a^.

Participant	DLQI_T0_^b^	DLQI_T1_^c^	Usage period (days)^d^	Difficulty^e^	Further use^f^	Severity level^g^	PASI^h^ score
1	1	1	3	A little	Not at all	Mild	Not replied
2	5	3	12	Fairly	Not replied	Heavy	Not replied
3	1	0	4	A lot	Not at all	Heavy	Not replied
4	9	19	6	A little	Not at all	Mild	0
5	7	2	21	Not at all	Regularly	Mild	Not replied
6	2	2	4	Fairly	Not at all	Moderate	Not replied
7	1	4	8	Not at all	Not at all	Moderate	<3
8	20	17	5	A little	As needed	Moderate	Not replied
9	15	9	5	Not at all	Regularly	Heavy	Not replied
10	10	12	3	Not at all	Not at all	Moderate	Not replied
11	15	—^i^	—	—	—	—	—
12	15	—	—	—	—	—	—
13	4	—	—	—	—	—	—
14	10	—	—	—	—	—	—
15	10	—	—	—	—	—	—

^a^Average values for participants 1 to 10: DLQI_T0_, 7.1, DLQI_T1_, 6.9, and usage period (days), 7.1. Average DLQI_T0_ value for participants 1 to 15 was 8.3.

^b^DLQI_T0_: Dermatology Life Quality Index before using the app.

^c^DLQI_T1_: Dermatology Life Quality Index after using the app.

^d^Indicates the number of days the app was used.

^e^Represents the difficulties experienced when using the app.

^f^Represents further use of the app.

^g^Shows the subjective perception of the severity of psoriasis.

^h^PASI: Psoriasis Area and Severity Index.

^i^Indicates participants who only completed the first questionnaire but did not withdraw their agreement.

**Qualitative feedback of participants. All statements were translated from German. Additions for better understanding are shown in square brackets. Detailed information on date, time, and medication are omitted because of privacy reasons.**

**Person A**

- “I have psoriatic arthritis and unfortunately the app does not relate to that.”
- “And unfortunately, I also have to say that I find the handling very complicated.”
- “Basically, I find the idea good, but so [the app] is unfortunately not further usable for me.”

**Person B**

- “It worked great.”
- “The “problem” is the photos. I live alone and find it difficult to photograph the back. But that is my only issue at the moment.”

**Person C**

- “I have tried a few apps, but they were all not applicable. Maybe theirs is a little better and more supportive.”

**Person D**

- “Also, I found it inconvenient and difficult to use at times because there was no explanation whatsoever.”

**Person E**

- “The “app” is not a native iOS or Android app, but a web application. I prefer here, especially for my sensitive health data, an app running locally on the iPhone/iPad, where the data is local on the device or encrypted in my own or the iCloud (optional).”

- “Using the app in parallel from multiple devices (smartphone, tablet, smartwatch) would be of great benefit.”

- “Such an app would also have to be individually “customizable” for me: Creation of own “therapies”. For the individual therapies, storage of more details (eg, for light therapy, the duration of the respective irradiation or the set Joule dose), for ointments, the name, PZN, etc.”
- “Usability would also have to be significantly optimized. For regular documentation (skin condition, condition, medication), the [documentation] must happen as quickly and easily as possible.”

**Person F**

- “The app is very interesting and helpful for people who don't have this background knowledge.”
- “The documentation via photos, I think is very good, I could have used that a lot from ***.”

**Person G**

- “I only used the app for a very short time, as I perceived filling it out as annoying.”
- “However, I think small changes to the app could fix this for the most part.”

**Person H**

- “Since I don't do therapy other than *** and *** and don't yet know what things help me, it would have also become difficult for me to use it meaningfully.”
- “I'm just looking more the other way around for a template where I can document what I've done and eaten (how much sleep, how much sun, etc) to figure out what factors are negatively impacting me.”

### Statistical Analysis

The targeted sample size (N=27) was not achieved. Therefore, the testing of the hypotheses was disregarded.

## Discussion

### Requirements Engineering and SMT Development

A special feature of the development process was the high level of participation. Community members were asked about additional features and could comment on the designs and mock-ups. Unfortunately, it was not possible to implement all the desired functions (see Table 1). Reminders could not be implemented due to the technical restriction of a PWA. Notifications are possible with this design but cannot be individually configured. Ratings of doctors and exchanges with other patients were not implemented, as these functions would overlap with the internet-based forum of the community. Some suggestions of the stakeholders have been partially implemented because the documentation should be in a structured form instead of a free form to avoid incorrect entries and simplify usage.

When examining for possible confounders, the usability aspects stood out. This was reinforced by the feedback from the individual participants. There were difficulties in dealing with the app. This was partly due to the handling and partly because patients have a greater need to document their illness than that assumed. In particular, the behavior for which therapy is currently being provided and secondary diseases should be documented more precisely and as quickly as possible. The heterogeneity of the users’ feedback shows that the app should be highly customizable. The suggestion to implement the app as a standalone one rather than as a PWA certainly warrants further IT security requirements and offers possibilities for including more features.

The feedback from the community shows a certain ambivalence regarding the privacy aspect. On the one hand, previous experience and feedback on the app show that a high level of data protection is desired. Therefore, all data remain locally stored on the user’s device. On the other hand, the fact that the data cannot be accessed on several devices was perceived as a negative feature. Apparently, there are services that enjoy a higher level of trust regarding data protection than others. Further developments to the app must ensure that users can decide whether they want to use the SMT data on 1 or more devices.

Despite the low number of study participants, the project shows the possibility of using citizen science in biomedical projects and research. The community-supported software development process successfully led to a functional SMT. In particular, the internet-based community provided the list of complementary measures, which were used as textual content in the SMT. Therefore, the SMT users could benefit from the collected patient knowledge about complementary measures without the effort of searching information on the internet.

### Limitations

There are 2 major limitations of this project. The first is the low overall sample size and the second is a small observed effect.

For showing a statistically significant improvement of a minimum of 4 score points, 27 participants would have been necessary for the study. This number was not reached despite previous experience about the active internet-based community and high involvement in the requirement engineering process. Only 21 participants could be recruited. Of these, only 10 participants completed all questionnaires. We identified potential reasons for this: (1) failure to reach the target group via social media channels, (2) an admission process that was perceived as too complicated, and (3) the Christmas holidays. One indication of this is an increase in the dropout rate in the month of December. The additional leaflets sent in December could not mitigate the low involvement.

The observed effect of 0.2 score points reported in the results is negligible. The fact that the desired effect with a difference of 4 DLQI points could not be measured can be attributed to different causes. Besides the low effectiveness of the intervention, the intervention period (21 days) may play a role. The reason is that regular use over a long period of time may be necessary to effect changes. Therefore, a measured effect could be considered a placebo effect. In addition, the DLQI was recorded without considering the occurrence of relapses, possible rehabilitation stays, or an existing concomitant disease such as psoriatic arthritis. Comparing the average DLQI score determined in this study with the results of the study by Augustin et al [46] (mean 7.5, SD 6.4 points) reveals that the values do not differ much. Moreover, the variances in the respective samples are similar. This difference of less than 1 score point with the score in this study is noteworthy because Augustin et al included not only patients of dermatology outpatient hospital clinics but also patients of dermatologists in ambulant care. In this study, the participants were recruited via the community, which is why it is unlikely that the sample consists exclusively of dermatology outpatient hospital clinic patients.

### Future Directions

Based on the feedback provided by the community and app users, the existing smartphone app should be completely revised. The authors strongly recommend developing such an app using a community-driven process again.

The improvements to the subsequent versions of the SMT can be roughly divided into four areas: (1) therapies including conventional medical therapies, hospital and rehabilitation stays, and complementary measures; (2) psoriasis relapses and triggers that can be analyzed; (3) behavioral patterns or environmental data in everyday life; and (4) QoL, as well as psychological and physical factors measured textually or visually using questionnaires.

To implement SMTs sustainably in care, it should be better integrated into the existing structures of care processes and medical practices. Direct data transmission to practitioners and further development of SMTs as approved medical products are conceivable.

### Conclusions

The high participation of the internet-based community in the development process and the response to the first survey (N=97) shows a general need for independently developed SMTs for people with psoriasis. However, the collected feedback shows that the solution presented in the paper does not meet the needs of the community. The authors conclude that a more customizable app is needed.

## Appendix

Multimedia Appendix 1 Semistructured German questionnaire and English translation used in the requirement engineering process.

Multimedia Appendix 2 Original German questionnaire and English translation for usability and acceptance of the self-management tool.

Multimedia Appendix 3 Study information website with information on the project that can be downloaded as a PDF file.

Multimedia Appendix 4 Informed consent of participants.

Multimedia Appendix 5 List of complementary measures suggested in the app, considering interactions.

## Abbreviations

DLQI: Dermatology Life Quality Index
PASI: Psoriasis Area and Severity Index
PSOAG: Psoriasis Selbsthilfe Arbeitsgemeinschaft eV (Psoriasis Self-Help Association)
PWA: progressive web app
QoL: quality of Life
SMT: self-management tool
