# Supplementary material for: A German Smartphone-Based Self-management Tool for Psoriasis: Community-Driven Development and Evaluation of Quality-of-Life Effects
Source: JMIR Form Res. 2022 Jul 7;6(7):e32593. doi: 10.2196/32593 (PMC9305401; doi:10.2196/32593)
Supplement: Multimedia Appendix 1 [file formative_v6i7e32593_app1.docx]

# Multimedia Appendix 1

Semi-structured German questionnaire and English translation used in the requirements engineering process.

## German version:

1. Wie stehst du einer solchen App gegenüber? Würdest du die App verwenden?

- Ja
- Nein, da ich kein Vertrauen in eine solche App habe.
- Ich würde die App auf jeden Fall testen.

2. Welche zusätzlichen Funktionen sollte eine solche App haben?

- Bei den Tagebucheinträgen soll es möglich sein, ein Foto zu hinterlegen.
- Der Verlauf der Schwere meiner Beschwerden soll erfasst werden können.
- Auf eigenen Wunsch will ich ausgewählte Daten mit meinem Arzt teilen.

3. Welche zusätzlichen Funktionen würdest du dir wünschen?

## English translation:

1. How do you feel about such an app? Would you use the app?

- Yes
- No, as I have no confidence in such an app.
- I would definitely test the app.

2. Which additional functions should such an app have?

- It should be possible to add a photo to the diary entries.
- It should be possible to record the progress of the severity of my complaints.
- I want to be able to share selected data with my doctor at my own request.

3. Which additional functions would you like to see?
