# Supplementary material for: A German Smartphone-Based Self-management Tool for Psoriasis: Community-Driven Development and Evaluation of Quality-of-Life Effects
Source: JMIR Form Res. 2022 Jul 7;6(7):e32593. doi: 10.2196/32593 (PMC9305401; doi:10.2196/32593)
Supplement: Multimedia Appendix 2 [file formative_v6i7e32593_app2.docx]

# Multimedia Appendix 2

Original German questionnaire and English translation for usability and acceptance of the self-management tool.

## German version:

**Fragebogen zur Verwendung der Psoriasis App**

Pseudonym: ______________ Datum: |__|__|__|

|  | An wie vielen Tagen haben Sie die App verwendet? | ____________ | /21 |  |
| --- | --- | --- | --- | --- |
|  | Wie schwer fiel Ihnen der Umgang mit der Psoriasis App? | sehr  ziemlich  ein bisschen  überhaupt nicht | 🞎  🞎  🞎  🞎 |  |
|  | Würden Sie die App nach der Studie weiterverwenden? | täglich  regelmäßig  nach bedarf  überhaupt nicht | 🞎  🞎  🞎  🞎 |  |
|  | Wie schwerwiegend schätzen Sie ihre Psoriasis ein? | schwer  mittelschwer  leicht | 🞎  🞎  🞎 |  |
|  | Bitte nur beantworten falls Sie ihren PASI-Score kennen und angeben wollen.  Wie hoch ist Ihr PASI-Score? | ____________ |  | Frage betrifft mich nicht 🞎 |

## English translation:

**QUESTIONNAIRE FOR THE USE OF THE PSORIASIS APP**

Pseudonym: ______________ Date: |__|__|__|

|  | On how many days did you use the app? | ____________ | /21 |  | |
| --- | --- | --- | --- | --- | --- |
|  | How difficult was it for you to use the Psoriasis App? | a lot  fairly  a little  not at all | 🞎  🞎  🞎  🞎 |  | |
|  | Would you continue to use the app after the study? | daily  regularly  as needed  not at all | 🞎  🞎  🞎  🞎 |  | |
|  | How severe do you consider your psoriasis to be? | heavy  mild  moderate | 🞎  🞎  🞎 |  | |
|  | Please only answer if you know your PASI score and want to report it.  How high is your PASI score? | ____________ |  | Question does not concern me | 🞎 |
