# Supplementary material for: A German Smartphone-Based Self-management Tool for Psoriasis: Community-Driven Development and Evaluation of Quality-of-Life Effects
Source: JMIR Form Res. 2022 Jul 7;6(7):e32593. doi: 10.2196/32593 (PMC9305401; doi:10.2196/32593)
Supplement: Multimedia Appendix 3 [file formative_v6i7e32593_app3.pdf]

Vielen Dank für Interesse an der Psoriasis-Studie. Ziel der Studie ist es zu untersuchen, ob eine Selbstmanagement-App für Psoriasispatient/-innen die Lebensqualität verbessern kann.
Bitte lesen Sie sich die folgenden Informationen gründlich durch.

Evaluation der Lebensqualität bei der Anwendung einer Selbstmanagement-App für Psoriasispatienten

Welches Ziel verfolgt die Studie?

Die Studie untersucht die Veränderung der Lebensqualität durch die Anwendung der bereitgestellten Selbstmanagement-App für Psoriasispatient/-innen.

Wie läuft die Studie ab?

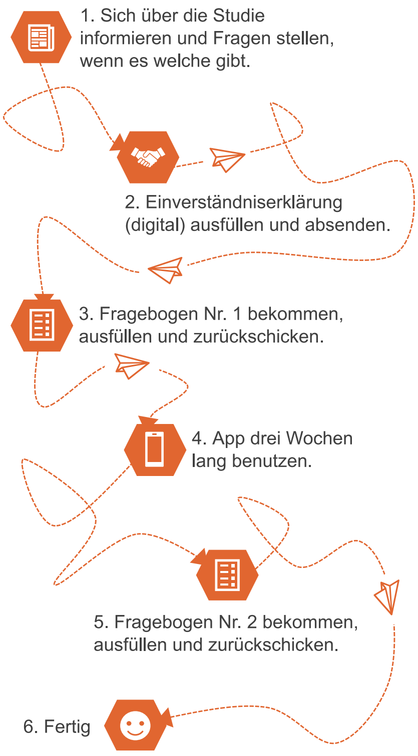

Die Anmeldung zur Studie erfolgt per formloser E-Mail an psoriasis-studie@hs-heilbronn.de.

- Bei Anmeldung zur Studie wird ebenfalls per E-Mail eine elektronische Einwilligung erteilt. Als nächstes erhalten Sie einen auszufüllenden Fragebogen, der zur Messung der Lebensqualität verwendet wird. Dieser wird als E-PDF ausgefüllt und per E-Mail zurückgesendet.
- Im Anschluss bekommen Sie die Selbstmanagement-App zur Verfügung gestellt, welche drei Wochen von Ihnen genutzt werden soll.
- Nach Ablauf dieser drei Wochen wird ein zweiter Fragebogen zur Lebensqualität, zur Benutzung der App, sowie des Krankheitszustands zugesandt. Dieser wird ebenfalls per E-Mail ausgetauscht.

Zur Identifikation der Fragebögen werden selbstgenerierte Pseudonyme verwendet. Direkt personenbezogene Daten werden in keinem der Fragebögen erhoben.
Die Studie erstreckt sich über einen Zeitraum vom 20.10.19 bis voraussichtlich 20.06.20.
An der Hochschule Heilbronn wurde ausschließlich für den Zweck Studie ein E-Mail Konto eingerichtet, welches nach Ablauf der Studie unwiderruflich gelöscht wird.
Dieses E-Mail-Konto dient der Anmeldung und dem Austausch der Fragebögen.

Wie beteilige ich mich hierdurch an Forschung?

Durch Ihre Teilnahme unterstützen Sie mich im Rahmen meiner Masterarbeit in der Medizinischen Informatik. Sie und andere Patient/-innen können von den Ergebnissen meiner Studie zukünftig profitieren.

Welche Risiken sind mit der Teilnahme verbunden?

Die in der Studie untersuchte App ist kein Ersatz für eine vom Arzt verordnete Therapie oder eine andere von Ihrem Arzt empfohlene Beratung. Ziel, der in der Studie verwendeten App, ist es speziell für Psoriasis-Patient/-innen ein Tagebuch bereitzustellen. In diesem führen Sie oder andere Patient/-innen Buch über ihre spezifischen Beschwerden, die durch ergänzende Maßnahmen gelindert werden sollen.

Die vorgeschlagenen Maßnahmen werden auf Wechselwirkungen überprüft. Es kann dennoch vorkommen, dass sich eine Maßnahme nicht für Sie eignet und es zu unerwarteten Nebenwirkungen kommen könnte. In diesem Falle ist von einer (weiteren) Anwendung der Maßnahme abzusehen.

Gibt es Ausschlusskriterien?

Für die Studie werden nur Personen über 18 Jahren zugelassen. Personen unter 18 Jahren können daher nicht teilnehmen.
Die Studie richtet sich ausschließlich an Patienten, die unter Psoriasis leiden. Patienten, die nicht an einer Form der Psoriasis leiden, können nicht an der Studie teilnehmen.

Informationen zum Datenschutz

Die Studienleitung wird alle angemessenen Schritte unternehmen, um den Schutz Ihrer Daten gemäß den deutschen Datenschutzstandards zu gewährleisten. Die Daten sind gegen unbefugten Zugriff gesichert.

- Während der Studie werden persönliche Informationen von Ihnen erhoben. Im Zusammenhang der Kontaktaufnahme und Korrespondenz werden personenbezogene Daten, wie z.B. Ihre E-Mailadresse, im E-Mail-Postfach der Hochschule Heilbronn elektronisch gespeichert. Das Postfach wird alleinig für die Studie verwendet und nach Beendigung der Studie unwiderruflich gelöscht. Ein Zugriff auf die darin enthaltenen Daten ist danach nicht mehr möglich.
- Die Studienergebnisse in Form der von Ihnen ausgefüllten Fragebögen werden an der Hochschule Heilbronn pseudonymisiert in Papierform aufbewahrt. Das auf den Fragebogen angegebene Pseudonym wird von Ihnen selbst generiert und ist von keiner anderen Person auf Sie zurückzuführen.
- Sobald das oben genannte E-Mail-Postfach gelöscht wurde, ist keine Zuordnung zwischen Fragebogen und E-Mailadresse mehr möglich.
- Die während der Studie erhobenen Daten, d.h. die Fragebögen, werden ausgedruckt und 10 Jahre in Papierform aufbewahrt. Die digitalen Fragebögen (E-PDFs) werden nach der Studie vernichtet.
- Die Daten werden ausschließlich zu Zwecken dieser Studie verwendet.
  Sie haben das Recht, vom Verantwortlichen (s.u.) Auskunft über die von Ihnen gespeicherten personenbezogenen Daten zu verlangen. Ebenfalls können Sie die Berichtigung unzutreffender Daten sowie die Löschung der Daten oder Einschränkung deren Verarbeitung verlangen.

Der Verantwortliche für die studienbedingte Erhebung personenbezogener Daten ist:
Frau Lea Brandl
Masterstudiengang Mezinische Informatik
Hochschule Heilbronn
Max-Planck Str. 39
74081 Heilbronn
E-Mail: psoriasis-studie@hs-heilbronn.de

Im Falle einer rechtswidrigen Datenverarbeitung haben Sie das Recht, sich bei folgender Aufsichtsbehörde zu beschweren:
Der Landesbeauftragte für den Datenschutz und die Informationsfreiheit Baden-Württemberg
Postfach 10 29 32, 70025 Stuttgart
Königstraße 10a, 70173 Stuttgart
Tel.: 0711/61 55 41 – 0
Fax: 0711/61 55 41 – 15
E-Mail: poststelle@lfdi.bwl.de
Internet: http://www.baden-wuerttemberg.datenschutz.de

Freiwilligkeit / Rücktritt & Widerspruch

Die Teilnahme an der Studie erfolgt freiwillig. Falls Sie teilnehmen möchten, bitten wir Sie, die beiliegende Einwilligungserklärung zu unterschreiben. Sie können diese Einwilligung jederzeit schriftlich oder mündlich ohne Angabe von Gründen widerrufen, ohne dass Ihnen dadurch Nachteile entstehen.
Wenn Sie Ihre Einwilligung widerrufen möchten, wenden Sie sich bitte an die oben angegebene E-Mailadresse. Bei einem Widerspruch können Sie entscheiden, ob die von Ihnen studienbedingt erhobenen Daten gelöscht werden sollen oder weiterhin für die Zwecke der Studie verwendet werden dürfen. Auch wenn Sie einer weiteren Verwendung zunächst zustimmen, können Sie nachträglich Ihre Meinung noch ändern und die Löschung der Daten verlangen; wenden Sie sich dafür bitte ebenfalls an die oben angegebene E-Mailadresse.
Beachten Sie, dass Daten, die bereits in wissenschaftliche Auswertungen eingeflossen sind oder Daten die bereits anonymisiert wurden, nicht mehr auf Ihren Wunsch gelöscht werden können.

Entstehen mir durch die Teilnahme Kosten oder erhalte ich eine Bezahlung bzw. Aufwandsentschädigung?

Die Studienteilnahme ist für Sie kostenlos. Sie erhalten allerdings auch keine Bezahlung.
Für weitere Informationen sowie für Auskünfte über allgemeine Ergebnisse und den Ausgang der Studie wenden Sie sich an Frau Lea Brandl (Tel: 017 ******* , E-Mail: psoriasis-studie@hs-heilbronn.de) zur Verfügung.
