# Supplementary material for: A German Smartphone-Based Self-management Tool for Psoriasis: Community-Driven Development and Evaluation of Quality-of-Life Effects
Source: JMIR Form Res. 2022 Jul 7;6(7):e32593. doi: 10.2196/32593 (PMC9305401; doi:10.2196/32593)
Supplement: Multimedia Appendix 4 [file formative_v6i7e32593_app4.pdf]

Evaluation der Lebensqualität bei der Anwendung einer Selbstmanagement App für Psoriasis Patienten

Einverständniserklärung

Ich habe die Informationsschrift gelesen und wurde schriftlich über das Ziel und den Ablauf der Studie sowie über die Risiken ausführlich und verständlich aufgeklärt. Ich hatte Gelegenheit telefonisch oder per Mail Fragen zu stellen. Alle meine Fragen wurden zu meiner Zufriedenheit beantwortet. Ich stimme der Teilnahme an der Studie freiwillig zu. Für meine Entscheidung hatte ich ausreichend Zeit. Ein Exemplar der Informationsschrift und der Einwilligungserklärung habe ich heruntergeladen und sorgfältig durchgelesen.

Ich bestätige hiermit, dass ich an einer Form der Psoriasis leide.

Geschäftsfähigkeit

Ich bestätige hiermit, dass ich das 18. Lebensjahr bereits begonnen habe und meine Geschäftsfähigkeit aus keinem weiteren Grund eingeschränkt ist.

Datum Unterschrift

Datenschutz

Mir ist bekannt, dass bei dieser Studie personenbezogene Daten verarbeitet werden sollen. Die Verarbeitung der Daten erfolgt nach gesetzlichen Bestimmungen und setzt gemäß Art. 6 Abs. 1 lit. a der Datenschutz-Grundverordnung folgende Einwilligungserklärung voraus:

Ich wurde darüber aufgeklärt und stimme freiwillig zu, dass meine in der Studie erhobenen Daten, insbesondere Angaben über meine Gesundheit, zu den in der Informationsschrift beschriebenen Zwecken in pseudonymisierter Form aufgezeichnet und ausgewertet werden können.

Dritte erhalten keinen Einblick in personenbezogene Unterlagen. Bei der Veröffentlichung von Ergebnissen der Studie wird mein Name ebenfalls nicht genannt. Die personenbezogenen Daten werden anonymisiert, sobald dies nach dem Forschungszweck möglich ist. Die Daten werden 10 Jahre in Papierform aufbewahrt. Daten in digitaler Form werden nach der Studie vernichtet.

Mir ist bekannt, dass diese Einwilligung jederzeit schriftlich ohne Angabe von Gründen widerrufen werden kann, ohne dass mir dadurch Nachteile entstehen. Die Rechtmäßigkeit der bis zum Widerruf erfolgten Datenverarbeitung wird davon nicht berührt. In diesem Fall kann ich entscheiden, ob die von mir erhobenen Daten gelöscht werden sollen oder weiterhin für die Zwecke der Studie verwendet werden dürfen.

Datum Unterschrift
