# Supplementary material for: A German Smartphone-Based Self-management Tool for Psoriasis: Community-Driven Development and Evaluation of Quality-of-Life Effects
Source: JMIR Form Res. 2022 Jul 7;6(7):e32593. doi: 10.2196/32593 (PMC9305401; doi:10.2196/32593)
Supplement: Multimedia Appendix 5 [file formative_v6i7e32593_app5.docx]

# Multimedia Appendix 5

List of complementary measures suggested in the app, considering interactions.

1. Relaxation exercises
2. Rubbing with an oil
3. Compresses
4. Removing flakes with plastic gloves or plastic wrap
5. Avoid pork
6. Partial baths for hands or feet
7. Curd compresses
8. Baths with salt
9. Reduce or avoid alcohol
10. Bath with medicinal bath additive (emulsion)
11. Use shower oil
12. Brine baths
13. Skin care with shea butter
14. Have your zinc levels checked
15. Seaside holidays
16. Have your vitamin D levels checked
17. Have your vitamin B12 levels checked
18. Have magnesium or vitamin B3 levels tested
19. Have thyroid checked
20. Folic acid
21. Skin care (general)
22. Sport and exercise
23. Integrate physical activity into your daily routine
24. Healing clay (external use)
25. Bath with oil (home remedy)
26. Oil pack for the head
27. Bath with medicinal bath additive (spreading bath)
28. Avoid hot spices, citrus fruits, and sour foods
29. Find (and go to) a self-help group
30. Maintain or revive a hobby
31. Do fasting
32. Seek psoriasis training
33. Descale with Loyon, Saalux head oil or similar products
34. Gluten-free diet
35. Check clothing: Does anything chafe?
36. Reduce or stop smoking
37. Go to the dentist to check for inflammation
38. Black tea poultice
39. Skin care with ghee
40. Make your own aloe vera care
41. Take short lukewarm showers instead of long hot baths
42. Visit the sauna
43. Meeting friends
44. Put a note on the mirror as a reminder
45. Skin care with urea
46. Dissolve flakes with salicylic acid
47. Skin care with a home-made skin care cream
48. Healing clay (internal use)
49. Mahonia aquifolium
50. Birch cork
51. Mediterranean diet
52. Omega-3 fatty acids
53. Practise the Mind-Body-Medicine method
54. Adjust nutrition
55. Cut nails short
